# A lncRNA Perspective into (Re)Building the Heart

**DOI:** 10.3389/fcell.2016.00128

**Published:** 2016-11-09

**Authors:** Stefan Frank, Aitor Aguirre, Juergen Hescheler, Leo Kurian

**Affiliations:** ^1^Cologne Excellence Cluster on Cellular Stress Responses in Aging-Associated Diseases, University of CologneCologne, Germany; ^2^Institute for Neurophysiology, University of CologneCologne, Germany; ^3^Center for Molecular Medicine (CMMC), University of CologneCologne, Germany; ^4^Division of Cardiovascular Medicine, Department of Medicine, University of California San DiegoLa Jolla, CA, USA

**Keywords:** long non-coding RNAs, embryonic development, homeostasis, cardiac development, cardiac regeneration, cell-fate commitment

## Abstract

Our conception of the human genome, long focused on the 2% that codes for proteins, has profoundly changed since its first draft assembly in 2001. Since then, an unanticipatedly expansive functionality and convolution has been attributed to the majority of the genome that is transcribed in a cell-type/context-specific manner into transcripts with no apparent protein coding ability. While the majority of these transcripts, currently annotated as long non-coding RNAs (lncRNAs), are functionally uncharacterized, their prominent role in embryonic development and tissue homeostasis, especially in the context of the heart, is emerging. In this review, we summarize and discuss the latest advances in understanding the relevance of lncRNAs in (re)building the heart.

“*His very existence was improbable, inexplicable, and altogether bewildering. He was an insoluble problem. It was inconceivable how he had existed, how he had succeeded in getting so far, how he had managed to remain – why he did not instantly disappear.”*

—Joseph Conrad, Heart of Darkness.

Over the last 20 years, the advent of novel sequencing technologies and initiatives such as ENCODE have revealed that the majority of the mammalian genome, despite not being translated into proteins, is pervasively transcribed into myriad RNAs of different sizes and characteristics, collectively referred to as non-coding RNAs (ncRNAs). To date, thousands of ncRNAs have been described in humans, yet the precise functional roles of a great majority remain unclear and controversial. Non-coding RNAs are traditionally classified based on their size into two major classes, small non-coding RNAs and long non-coding (lnc) RNAs. Small non-coding RNAs were first described in 1993 (Lee et al., 1993), and have been studied extensively since then, along with their molecular mechanisms of action and function. Although much is left to be learnt from them (e.g., piRNAs) they are outside the scope of this review, but readers can consult the excellent reviews (Bartel, 2004, 2009; Carthew and Sontheimer, 2009; Malone and Hannon, 2009; Mendell and Olson, 2012).

Long non-coding RNAs are defined as a large and diverse group of non-protein coding transcripts longer than 200 nucleotides (Rinn and Chang, 2012). Currently, they represent one of the most prominent but least understood genes in vertebrates. Overall, more than ~100,000 genomic loci are predicted to produce lncRNAs in the human genome (Zhao et al., 2016) and account for the largest class of genes, giving an idea of the potential relevance of this class of molecules. The consistent observation of cell type/tissue specific expression, together with their increased presence in evolutionarily more complex organisms, lncRNAs have been proposed to play key roles in affecting diverse cellular functions from modulating chromatin states to protein synthesis. Ultimately, this imparts a new layer of sophistication to the control of fundamental biological processes. In line with this, their roles in health and disease, including embryonic development, homeostasis, cancer, neurodegenerative, and metabolic disorders are rapidly becoming clear.

As evident from the exponentially increasing body of literature, lncRNAs are particularly interesting in the context of cardiac biology. The hierarchy and ontogeny of cardiac specification is becoming increasingly complex with the identification of primary, secondary [and potentially tertiary (Bressan et al., 2013)] heart fields that together give rise to the multitude of highly specialized cell types that constitute the mammalian heart [for review, see (Buckingham et al., 2005; Brade et al., 2013)]. Among multiple modes of gene regulation that fine tune cardiac development, recent studies reveal a prominent role for lncRNAs (Grote et al., 2013; Klattenhoff et al., 2013; Ounzain et al., 2014, 2015a,b; Kurian et al., 2015). Additionally, dysregulated lncRNAs have been implicated in cardiovascular diseases (Yap et al., 2010; Han et al., 2014; Michalik et al., 2014; Ounzain et al., 2014, 2015a,b; Wang et al., 2014a,b; Yan et al., 2015), including coronary artery and other heart dysfunctions, which remain the leading cause of mortality in the developed world, ahead of all cancer types combined. In the United States alone, 80 million people suffer from cardiovascular disorders, and cardiovascular diseases are projected to be the leading cause of death in the world by 2020, highlighting the importance of a better understanding of their etiology. Cardiac injuries, such as myocardial infarction (MI), usually caused by an occlusion of a coronary artery, can lead to immediate death due to the loss of oxygenation in the ventricular muscle. Such an occlusion quickly results in ischemia/reperfusion injury and necrosis of the tissue. Patients who survive a cardiac episode face progressive deterioration due to compensatory responses over several years, resulting in heart failure and eventually death. Although the molecular and cellular mechanisms involved in these pathologic responses are now better understood, many puzzles remain to be solved and the treatment of cardiac disorders remains poor. Investigating the regulatory functions of lncRNAs in cardiac homeostasis and disease might open new avenues in the treatment of cardiac disorders. In this review, we will summarize the latest progress in understanding the molecular mechanisms of lncRNA function in cardiac biology and their potential as novel therapeutic targets in the future.

## Evolution of the lncRNA paradigm—from junk to rosetta stone

The study of lncRNAs originates from the interest in the non-protein coding (hereafter termed non-coding) part of the genome, its evolution and its potential function in the 1950s. Scientists quickly discovered the *C*-value paradox, that is, the lack of correlation between organismal size or developmental complexity and the DNA content of a cell. Lower animals—from an anthropocentric perspective—such as salamanders and fish, can have a genome many times larger than higher order organisms, such as humans. The paradox seemed solved when it was found that most of the genome does not encode for protein-coding genes (Ohno, 1972). The non-coding portion of the genome was prematurely termed “junk DNA” due to the high presence of transposons, pseudogenes, repeats, and other elements of dubious or unknown function. Interestingly, this part of the genome accounts for 50–70% of the mammalian genome (de Koning et al., 2011). However, even then geneticists were still puzzled by the C-paradox. Although a part of the non-coding genome accounted for structural regions in the DNA, the majority remained unexplained. So the question remained: why would organisms maintain such a large genome—at high energy cost—without having any major function? Indeed, many of the early pioneers suspected something more was at stake and expressed their belief that the non-coding genome might not be entirely useless.

The first evidence for the potential function of the non-coding part of the genome was that the transcriptional activity in cells was too high to be entirely attributable to coding genes and ribosomal RNAs. Heterogeneous nuclear RNAs were soon discovered as RNAs originating from repetitive and heterochromatic regions with no protein-coding ability (Yunis and Tsai, 1978). Some years later, more non-coding RNAs involved in post-transcriptional regulation were found, including snRNAs and snoRNAs. However, it was not until the advent of microarrays and next-generation sequencing methodologies in the last decades that our picture of non-coding RNAs became more complete. The scale of pervasive transcription is much larger than previously appreciated, with estimates ranging from 70 to 90% of the human genome being transcribed at one point or another during development, homeostasis or disease (Bertone et al., 2004; Mercer and Mattick, 2013). Some of these transcripts are present at very low copy numbers and controversy still exists on their actual relevance. Indeed, a number of putative lncRNA transcripts might be the effect of pervasive transcription and transcriptional “noise.” However, detailed RNA-sequencing analysis and chromatin signatures (histone modifications, TF binding, DNase I hypersensitivity assays) indicate strong control of regulatory elements in these loci, suggesting that a significant percentage of lncRNA transcripts indeed constitute novel non-coding genes with currently unknown function (Guttman et al., 2009; Cabili et al., 2011; Iyer et al., 2015). It would seem now that ncRNAs play more important roles than anticipated and might constitute a “Rosetta Stone,” a new layer of gene regulation that might offer insight into the complexity of higher organisms.

## Classes of lncRNAs and their genomic architecture

**Figure 1:**
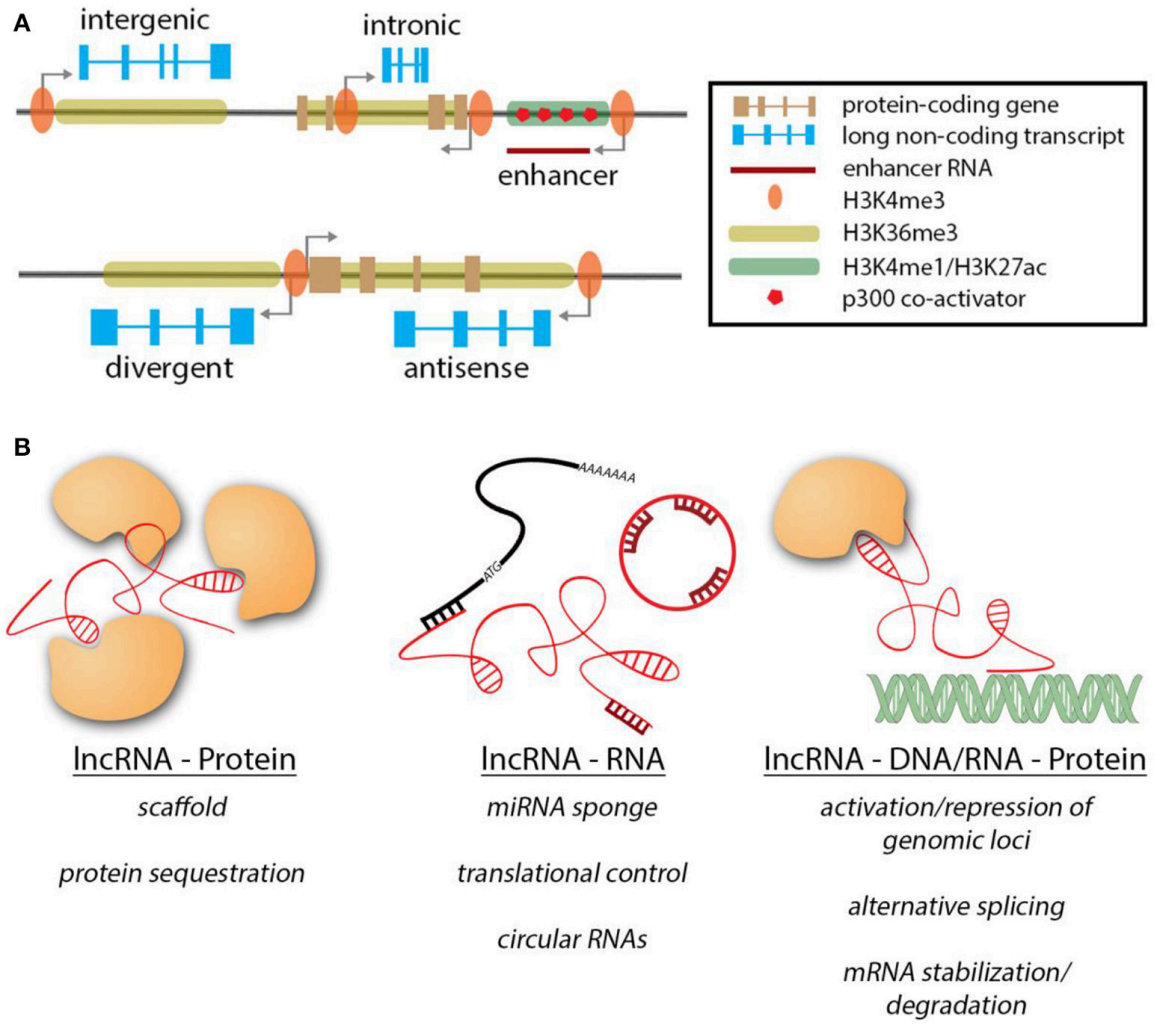
**Genomic contexts of lncRNAs & modes of action. (A)** LncRNA genes (blue) can exist in various genomic contexts. The histone marks are similar to protein-coding genes. NcRNAs transcribed from enhancer regions are unique in their features. **(B)** Overview of the different interactions of lncRNAs and their target molecules.

Being defined solely by size and a lack of coding potential, lncRNAs constitute a large and heterogeneous group of RNA molecules. It is therefore not surprising that their genomic contexts show a similar variability (Figure 1A; Cabili et al., 2011). Strikingly, a large fraction of lncRNA loci and transcripts show strong similarities to protein-coding transcripts with regards to histone marks occupying their promoters and gene body, intron-exon structure and poly-adenylation. Based on their genomic architecture, lncRNAs are categorized as long intergenic non-coding RNAs (lincRNAs), antisense lncRNAs, intronic lncRNAs, circular RNAs, divergent lncRNAs, and enhancer RNAs. A major class of lncRNAs, long intergenic non-coding RNAs (lincRNAs), is located in barren gene deserts or between two protein-coding genes. This particular class operates as stand-alone genes, often having their own regulatory landscape. Another class of lncRNAs, intronic lncRNAs, is located within introns of protein-coding genes similar to what has been reported for many microRNAs. Antisense lncRNAs are transcribed from the opposite strand of a protein-coding RNA. They can also exist outside the gene body of their associated coding gene, resulting in various degrees of transcriptional overlap, ranging from short stretches to a complete overlap. A different form of antisense transcripts is divergent lncRNAs. These genes are controlled by the same promoter region as the coding neighbor, but are transcribed in the opposite direction to the coding gene, without any transcriptional overlap. The function of this class of lncRNAs is very poorly understood. In contrast, enhancer RNA (eRNA) and circular RNAs (ciRNAs) are uniquely distinct in their genomic architecture. eRNAs are transcribed by active enhancers and are often single exonic and not poly-adenylated. Furthermore, during active transcription the gene body is not marked by H3K3me3, but H3K4 monomethylation and H3K27 acetylation in combination with abundant binding of the p300 co-activator (De Santa et al., 2010; Kim et al., 2010). The function of eRNA transcripts currently remains controversial. Recently, a novel class of lncRNAs arising from circularization of exons has been described (Zhang et al., 2013). Interestingly these circular RNAs (ciRNAs) can emerge from protein-coding genes by covalently linking separate exons.

## Molecular mechanisms of lncRNA-mediated gene regulation

Similar to the diverse genomic contexts in which lncRNA genes can be found, lncRNAs can exert their regulatory function through a wide variety of mechanisms (Figure 1B). In contrast to the well-studied microRNAs, lncRNAs are not limited to interactions with other transcripts, but can exert their function along with proteins, RNAs and DNA, thereby expanding the dimensions by which they can operate. When interacting with proteins, lncRNAs can act as guides for targeting a protein/protein complex to specific sites/subcellular locations (Rinn et al., 2007; Yap et al., 2010; Grote et al., 2013), as scaffolds to bring different subunits of protein complexes together and maintain them in close proximity (Wang et al., 2004; Tsai et al., 2010; Yang et al., 2011) or act as decoys to sequester proteins away from their site of action (Kino et al., 2010; Tripathi et al., 2010; Hung et al., 2011). In the context of the heart, lncRNA Myheart (Mhrt) directly binds to Brg1 in order to inhibit its helicase function (Han et al., 2014). Similarly, lncRNAs carrying repeats of corresponding complimentary sequences to miRNAs can sequester them acting as miRNA sponges [e.g., CARL (Wang et al., 2014b), CHRF (Wang et al., 2014a), HULC (Cui et al., 2015), linc-MD1(Legnini et al., 2014), H19(Giovarelli et al., 2014; Tao et al., 2016)]. A very recently discovered example of lncRNA-RNA interaction is mediated by circular RNAs that can act as miRNA sponges [e.g., ciRS-7(Xu et al., 2015; Geng et al., 2016; Yu et al., 2016)]. It remains to be determined if ciRNAs can impart their function via other mechanisms.

In addition, lncRNAs can regulate protein coding transcripts by controlling their translation [Uchl1-as (Carrieri et al., 2012), lincRNA-p21(Yoon et al., 2012)] or by regulating transcript stability. For example, the lncRNA TINCR binds Staufen 1 (Stau1) in order to stabilize mRNAs containing the TINCR binding motif (Kretz et al., 2013). Besides interacting with other RNA molecules, lncRNAs can also bind to DNA, often in complex with proteins, resulting in multi-layered regulatory complexity. Many cases of activation or repression of genomic loci by lncRNAs have been described to date [HOTAIR (Gupta et al., 2010), Braveheart (Klattenhoff et al., 2013), FENDRR (Grote et al., 2013)]. In this scenario, lncRNAs recruit epigenetic modifiers to target specific genomic loci, leading to either activation or inactivation. LncRNAs have also been shown to regulate gene activity co-transcriptionally, e.g., by aiding alternative splicing of mRNA isoforms (Gonzalez et al., 2015) or binding to newly synthesized mRNA, resulting in their stabilization or degradation (Matsui et al., 2008; Gong and Maquat, 2011; Wang et al., 2016a). eRNAs have been demonstrated to act in cis to regulate expression of target (protein-coding) genes (Ounzain et al., 2014). Another example for lncRNAs acting in cis is during genomic imprinting, an important process during development, where bi-allelic expression is changed to monoallelic and *viceversa*. Several lncRNAs have been demonstrated to mediate imprinting of neighboring genomic loci [Xist & Tsix (Brown et al., 1992; Clemson et al., 1996; Lee et al., 1999; Bell et al., 2014; Chu et al., 2015; McHugh et al., 2015), KCNQ1OT1 (Thakur et al., 2004), Airn (Sleutels et al., 2002)].

Recently, a handful of reports indicated that some lncRNAs work by more conventional means than previously expected. Several groups have independently demonstrated that a fraction of genes currently annotated as lncRNAs can be translated to code for short peptides (Chng et al., 2013; Pauli et al., 2014, 2015). This also explains why some lncRNAs appear preferentially located in the cytoplasm and in association with ribosomes in a manner similar to typical mRNAs. In an elegant study, Pauli et al. identified 28 candidate signaling proteins expressed during zebrafish embryogenesis, including toddler [(Pauli et al., 2015); also identified as ELABELA (Chng et al., 2013)], a previously annotated lncRNA which in reality codes for a short, conserved, secreted peptide. Impaired expression of Toddler reduces the motility of mesendodermal cells during zebrafish gastrulation and leads to grave developmental defects, including cardiovascular defects. Toddler promotes cell movement locally, suggesting that it is neither an attractant nor a repellent, but acts as a motogen. Toddler promotes the internalization of G-protein-coupled APJ/Apelin receptors and activation of APJ/Apelin signaling rescues toddler mutants. Intriguingly, toddler and similar short peptides might constitute a previously unrecognized family of signaling molecules crucial for vertebrate development. Recently, the Olson lab reported another short peptide (DWORF) translated from a previously annotated long non-coding RNA (Nelson et al., 2016). DWORF enhances muscle contractility by regulating Ca^2+^ levels in skeletal muscle cells. Based on these studies, the genes annotated as lncRNAs should be studied carefully to discriminate between a transcript specific function, a function by the act of transcription or potential peptides/novel proteins encoded by them. Taken together, lncRNAs regulate cellular events in impressively wide mechanistic avenues.

## Role of lncRNAs in modulating cardiac development

In recent years, lncRNAs have been extensively studied in both development and disease. The majority of novel lncRNAs have been associated to carcinogenesis and cancer progression. However, several studies have also demonstrated a crucial role for lncRNAs in development and disease, their contribution being particularly prominent in cardiac development and cardiac disorders (see Table 1). These findings probably reflect more the active efforts by the cancer and cardiac community to tackle these prominent diseases, rather than a biological overabundance of lncRNAs in cancer or cardiac-related cells. While thousands of lncRNAs have been reported to be expressed exclusively in the heart during development or disease, their function remains to be explored (Ounzain et al., 2014, 2015b).

**Table 1:** **Summary of lncRNAs involved in cardiac development and disease**.

**Name**	**Species**	**Genomic context**	**Function and mechanism**	**Location (human hg38, mouse mm10)**	**References**
ALIEN	Human	Intergenic	Cardiovascular commitment	chr20:22,560,553–22,578,642	Kurian et al., 2015
ANRIL	Human	Antisense	Metabolism, coronary artery disease, myocardial infarction	chr9:21994790–22121097	Yap et al., 2010; Vausort et al., 2014
Bvht	Mouse	Intergenic	Cardiac mesoderm commitment Decoy	chr18:61639653–61647503	Klattenhoff et al., 2013
Carl	Mouse	Intergenic	Mitochondria, cardiomyocyte apoptosis; miRNA sponge	chr2:18799244–18801291	Wang et al., 2014b
CARMEN	Mouse/Human	Intergenic	Cardiac specification & homeostasis; enhancer-associated, cis- & trans-regulation	chr5:149,406,845–149,432,836 (human) chr18:61,645,878–61,665,538 (mouse)	Ounzain et al., 2015a
CHAST	Mouse/Human	Antisense	Pro-hypertrophic; cis-regulation	chr17:64,783,199–64,783,552 (human) chr11:103,363,213–103,364,651 (mouse)	Viereck et al., 2016
Chrf	Mouse	Intergenic	Cardiac hypertrophy; miRNA sponge	chr18:72164057–72165898	Wang et al., 2014a
Fendrr	Mouse/Human	Intergenic	Cardiac development; cis- & trans-regulation, Guide	chr16:86,474,529–86,508,860 (human) chr8:121059119–121083032 (mouse)	Grote et al., 2013
H19	Mouse/Human	Antisense	Cardiac fibroblast proliferation, negative regulator of hypertrophy	chr11:1995176–1997835 (human) chr7:142575532–142578146 (mouse)	Tao et al., 2016
HIF1A-AS2	Human	Antisense	Myocardial infarction	chr14:61747039–61749089	Vausort et al., 2014
KCNQ1OT1	Human	Antisense	Cardiovascular development, arrhythmia, myocardial infarction; Guide	chr11:2,608,328–2,699,994	Thakur et al., 2004; Korostowski et al., 2012; Vausort et al., 2014
LIPCAR	Human	Unknown	Heart failure	chrM:7586–15887	Kumarswamy et al., 2014
Malat1	Mouse/Human	Intergenic	Endothelial cell identity, myocardial infarction; Decoy	chr11:65,497,762–65,506,469 (human) chr19:5,795,690–5,802,671 (mouse)	Michalik et al., 2014; Vausort et al., 2014
MEG3	Mouse/Human	Intergenic	Modulation of TGF-β pathway; Guide	chr14:100,826,134–100,861,008 (human) chr12:109542023–109568650 (mouse)	Mondal et al., 2015
Mhrt	Mouse/Human	Intergenic	Cardiac hypertrophy; Decoy	chr14:23,415,450–23,417,595 (human) chr14:54,968,787–54,974,349 (mouse)	Han et al., 2014
MIAT	Human	Intergenic	Myocardial infarction; miRNA sponge	chr22:26657482–26676477 (human) chr5:112220925–112228948 (mouse)	Yan et al., 2015
Mm67/77/85/130/132	Mouse	Intergenic	Cardiac development & remodeling; Enhancer-associated lncRNA Cis-regulation	Several loci (see original publication)	Ounzain et al., 2014
Novlncs	Mouse	Intergenic	Cardiac remodeling; Enhancer-associated	Several loci (see original publication)	Ounzain et al., 2015b
NRF	Mouse	Intergenic	Regulated necrosis of cardiomyocytes; miRNA sponge	chr3:45,438,398–45,440,956	Wang et al., 2016b
PANCR	Human	Intergenic	Cardiac differentiation	chr4:110,595,513–110,615,458	Gore-Panter et al., 2016
PUNISHER	Human	Antisense	Endothelial cell identity	chr12:57,726,240–57,728,356	Kurian et al., 2015
PVT1	Mouse	Antisense	Cardiomyocyte cell size, possibly regulating hypertrophy	chr15:62,037,986–62,260,212	Yu et al., 2015
RNCR3	Mouse/Human	Intergenic	Atheroprotective; miRNA sponge	chr13:90,060,247–90,119,719 (human) chr14:64,588,115–64,593,961 (mouse)	Shan et al., 2016
ROR	Human	Intergenic	Pro-Hypertrophic; miRNA sponge	chr18:57,054,559–57,072,119	Jiang et al., 2016
SENCR	Human	Antisense	Smooth muscle contractility	chr11:128,691,672–128,696,023	Bell et al., 2014
Smad7-lncRNA	Mouse	Antisense	Enhancer-associated lncRNA, Cis-regulation	chr18:75522879–75528680	Ounzain et al., 2014
SMILR	Human	Intergenic	Vascular smooth muscle cell proliferation	chr8:122,414,332–122,428,551	Ballantyne et al., 2016
TERMINATOR	Human	Intergenic	Pluripotency, cardiovascular development	chr1:200,411,800–200,475,513	Kurian et al., 2015
uc.167	Mouse	Antisense	Involved in cardiac differentiation	chr5:88,889,445–89,465,982	Song et al., 2016
UCA1	Human		Biomarker for acute myocardial infarction, anti-apoptotic (rat model)	chr19:15,828,947–15,836,321	Liu et al., 2015; Yan et al., 2016

**Figure 2:**
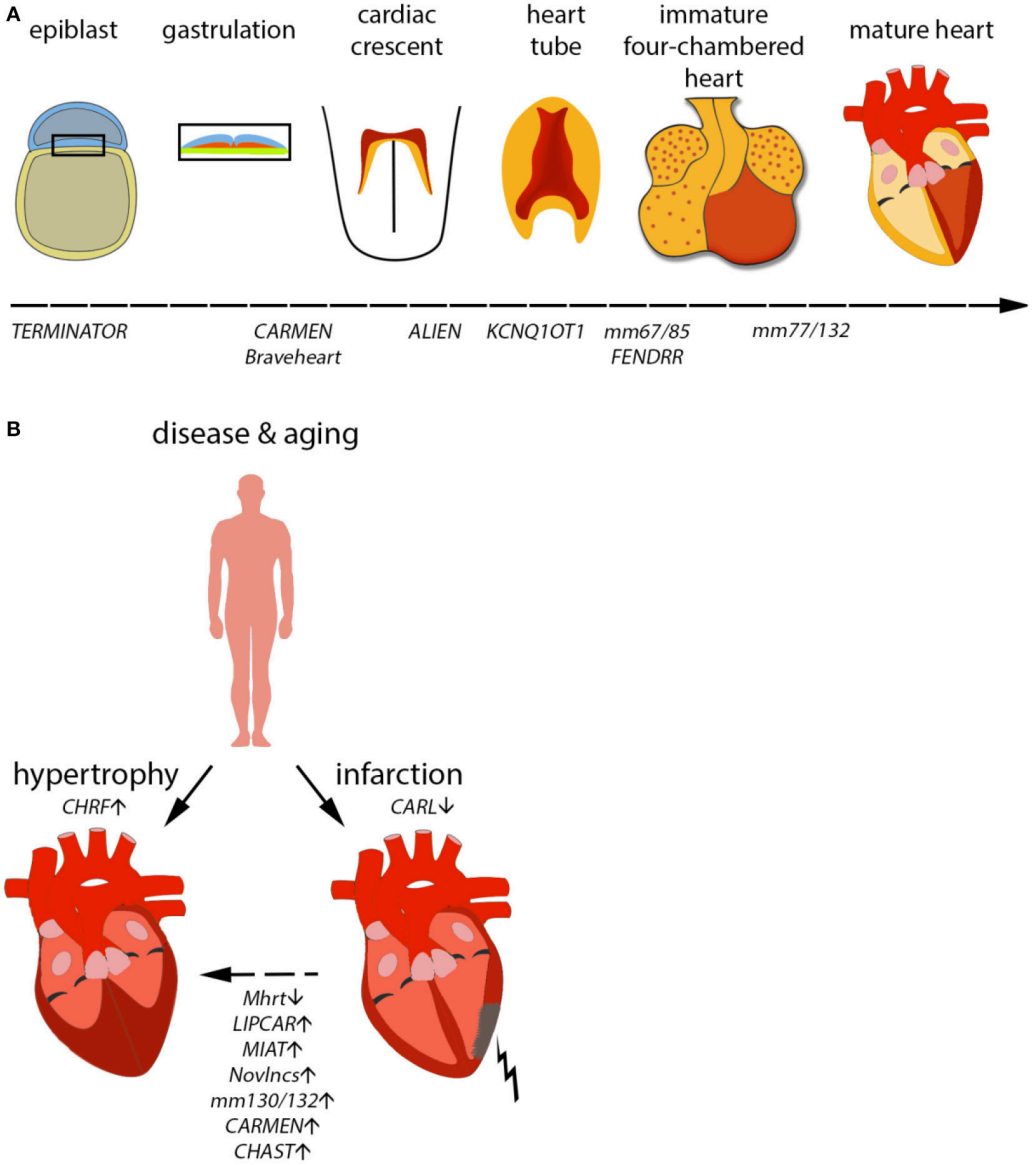
**Mammalian cardiac development ***in vivo/in vitro*** and consequences of aging on the heart. (A)** Development of the mammalian heart is a complex series of events, marked by distinct morphological changes. Expression of selected lncRNAs is indicated along the developmental timeline. **(B)** During aging, the heart becomes hypertrophic, i.e., the heart muscle increases by an increase in cell size, not number. Furthermore, the risk of myocardial infarction (MI) increases with age. MI can eventually result in hypertrophy in order to compensate for the loss in heart muscle. Selected lncRNAs identified to play a role in infarction or hypertrophy are depicted.

The heart is the first organ to form and function in an embryo, and survival of the embryo and all subsequent events strongly depends on its uninterrupted function. Heart development is an extraordinarily complex process involving the migration and integration of multiple cell lineages into a three-dimensional organ and its adequate connection with the vascular system (Figure 2A). Abnormalities in heart development lead to congenital heart disease, the most common human birth defect, which results in a series of functional defects and a host of potentially fatal disorders, including arrhythmias, cardiomyopathies, heart failure, and sudden death (Olson and Schneider, 2003; Bruneau, 2008).

Due to its importance in early survival, it is not surprising that the formation of the cardiovascular system and its function are precisely controlled by integrated networks of transcription factors that link signaling systems with the protein-coding genes required for cardiac myogenesis, morphogenesis, and contractility (Pipes et al., 2006). Surprisingly, until recently we were unaware that these pathways are intimately intertwined with dozens of critical non-coding genes, including microRNAs and long non-coding RNAs (Pipes et al., 2006; Aguirre et al., 2014; Kurian et al., 2015; see Table 1). These non-coding RNAs act as fine switches to modulate and choreograph multiple aspects of cardiac development, function, disease, and injury recovery. The increasing number of non-coding elements identified to date illustrates the importance of ncRNAs in modulating and creating complex phenotypes at the cellular and organ level, especially in the context of the heart.

The first lncRNA identified as a critical regulator of cardiac development was *Braveheart* (Klattenhoff et al., 2013). *Braveheart* controls the cardiac gene network in multipotent cardiac progenitors derived from mouse ESCs and functions upstream of mesoderm posterior basic helix-loop-helix transcription factor 1 (MESP1), a master regulator of cardiac development. *Braveheart* acts as an epigenetic regulator of cardiac commitment by interacting with SUZ12, a member of the polycomb repressive complex 2 (PRC2). As of now, it remains to be seen if *Braveheart* is required for normal heart development *in vivo*. Unfortunately, *Braveheart* seems to be a mouse-specific lncRNA and no homologs have been found in other species. Its expression is well-documented in mouse ESCs and heart samples using RNA sequencing. However, the orthologous human and rat genomic regions are not actively transcribed. The lack of a *Braveheart* homolog in humans begs the question of how well its knowledge will translate into the human cardiac setting. Given its critical importance in cardiac lineage commitment, it is however possible that an undiscovered functional *Braveheart* homolog gets transcribed from a different genomic locus.

Very few lncRNAs have been functionally interrogated *in vivo* in mammalian models. Fendrr, a novel lncRNA expressed in the heart, has been explored using mouse genetics. Two independent studies demonstrated that loss of *Fendrr* is embryonic lethal in mice (Grote et al., 2013; Sauvageau et al., 2013). Mutants display numerous developmental defects, including disrupted cardiac morphogenesis, consistent with the finding that *Fendrr* is expressed in the mouse lateral plate mesoderm and developing hearts. However, there is a difference in the phenotype severity associated with the two mutant mouse lines developed; while one mutant line dies at embryonic day E13.75(Grote et al., 2013), the other one dies postnatally (Sauvageau et al., 2013). One possible explanation for this discrepancy is the different targeting strategies used to remove the *Fendrr* gene. Mechanistically, *Fendrr* has been shown to interact with the PRC2 complex to modulate the epigenetic regulation of gene expression, and might be involved in the control of the activating H3K4me3 mark on a subset of promoters, thus modifying the expression level of those genes (Grote et al., 2013). However, the mechanisms of *Fendrr*-dependent molecular events remain to be fully understood.

Embryonic stem cell (ESC) based differentiation models provide a powerful tool for discovery and functional identification of novel long non-coding RNAs in humans. Current differentiation protocols allow for the precise *in vitro* recapitulation of the early developmental steps leading to cardiac commitment and cardiac or endothelial cell generation (Kurian et al., 2013; Bhattacharya et al., 2014; Burridge et al., 2014; Zhang et al., 2015). Using human ESCs and zebrafish as a model of vertebrate development, we recently were able to identify 76 novel human lncRNAs partially conserved across vertebrates, and characterized three of them in cardiovascular development in vertebrates (Kurian et al., 2015). These three lncRNAs, named TERMINATOR, ALIEN [also known as linc00261 and DEANR1(Jiang et al., 2015)] and PUNISHER, are essential at different stages of mesodermal and cardiovascular development, albeit their specific mechanisms of action still remain poorly understood. TERMINATOR is an abundant transcript in pluripotent stem cells and is essential for early embryonic survival, pluripotency, and early mesendodermal commitment both in zebrafish and mouse embryos. In contrast, ALIEN is more specifically expressed in mesendodermal tissues and its loss-of-function leads to disrupted vasculature and heart formation. Interestingly, ALIEN is also an endodermal lncRNA participating in pancreatic differentiation through its interaction with FOXA2 and SMAD2/3 (Jiang et al., 2015).

## Regulation of cardiac function and disease by lncRNAs in the adult

Besides their involvement in cell fate decisions and developmental programs, lncRNAs are also important regulators of homeostasis in the adult cardiovascular system, and their dysregulation appears associated with a variety of disease states.

### Chromatin remodeling and epigenetic state regulation by lncRNAs in the diseased heart

A prominent feature of the aging or failing heart is hypertrophy, a process involving a pathological increase in cardiomyocyte size in order to cope with inefficient cardiac function (Figure 2B). Two lncRNAs have been demonstrated to influence cardiac hypertrophy in murine models. The first of them was named Myheart (Mhrt, for myosin heavy chain-associated RNA transcript) and constitutes a cardioprotective lncRNA cluster transcribed antisense from the Myh7 locus in mouse (Han et al., 2014). Mhrt is specific to cardiac tissue and highly expressed in the heart. During pathological stress, such as pressure overload, Mhrt expression is silenced by a chromatin remodeling complex (Brg1-Hdac-Parp). This repression is an essential step in the progression of dilated cardiomyopathy. Restoring Mhrt expression to pre-stress levels is enough to protect from disease progression and heart failure. Interestingly, it seems that Mhrt antagonizes Brg1 (also known as Smarca4) directly by binding to its helicase domain and preventing Brg1 recognition of its DNA targets. A human version of *MHRT* exists and is repressed in cardiomyopathy cases, suggesting the same mechanism is present in humans. Another lncRNA involved in the same pathological process of heart remodeling and hyperthrophy is lncRNA CHRF (cardiac hypertrophy-related factor; Wang et al., 2014a). However, CHRF regulates hypertrophy by very different mechanisms compared to Mhrt. During hypertension, angiotensin-II production stimulates paracrine and hormonal release of pro-hypertrophic factors, such as endothelin-1 and TGF-β1. Under these conditions, microarray profiling experiments were able to identify microRNA-489 as a significantly downregulated transcript. Overexpression of this microRNA was enough to attenuate the hypertrophic phenotype in cardiomyocytes. Under cardiomyopathy conditions, lncRNA CHRF is expressed and acts as a sponge for microRNA-489, preventing it from exerting its function, which is the downregulation of Myd88 (a known inducer if cardiac hypertrophy). In another very promising recent study, the pro-hypertrophic lncRNA CHAST was identified (Viereck et al., 2016). CHAST was found to be functionally conserved and *in vivo* silencing of this lncRNA resulted in an improved recovery after cardiac injury, demonstrating a potential treatment of pathological heart remodeling processes.

### Cardiac metabolism, mitochondrial function, and lncRNAs

Metabolism is an essential aspect of cellular physiology, even more so in cardiomyocytes and the heart, an organ with an extraordinary bio-energetic demand. Metabolic remodeling is a hallmark of heart failure and cardiomyopathy and is characterized by a reversion to a fetal-like glycolytic metabolism and a capacity to efficiently employ fatty acid oxidation—the preferred energy source for the adult heart. Very few lncRNAs related to cardiac metabolism have been identified to date. CARL (cardiac apoptosis-related lncRNA) is a lncRNA identified during anoxic treatment of cardiomyocytes (Wang et al., 2014b). During anoxia, PHB2, an important regulator of mitochondrial fission and fusion dynamics, is strongly downregulated by the increased expression of miR-539, leading to mitochondrial fission and cardiomyocyte apoptosis. Interestingly, this dramatic upregulation of miR-539 is the direct consequence of lncRNA CARL silencing. CARL acts as a sponge for miR-539, and is highly expressed in the heart as well as other tissues, indicating this mechanism of apoptosis might be relevant outside the cardiac setting. Forced overexpression of CARL is sufficient to counter the effects of anoxia and rescue a significant proportion of cardiomyocytes from apoptosis. The trigger for CARL downregulation remains unknown.

Recently, a lncRNA profiling study carried out in plasma from patients suffering from heart failure identified a number of circulating lncRNAs associated with LV (left ventricular) remodeling (Kumarswamy et al., 2014). The authors were able to detect hundreds of lncRNAs, and in an interesting turn of events, found out that the most abundant majority of plasma lncRNAs originate from the mitochondrial genome (77%). One lncRNA was significantly upregulated in heart failure and predicted cardiac remodeling with high probability. This lncRNA, named LIPCAR (for long intergenic non-coding RNA predicting cardiac remodeling), increases significantly during late-stage post-myocardial infarction remodeling and during chronic heart failure, thus constituting a prognostic marker for heart failure. The mechanisms of action of LIPCAR and its function at the cellular and tissue level remain to be described.

### Control of hypertension and endothelial cell dysfunction by lncRNAs

ANRIL (antisense non-coding RNA in the INK4 locus) plays a critical role in hypertension and atherosclerosis (Yap et al., 2010). ANRIL was discovered accidentally by several independent genome-wide association studies (GWAS) indicating the presence of a coronary artery disease (CAD) risk locus in chromosome 9p21. No known protein-coding elements were present in the haplotype block, however a transcript of unknown function did originate and was very active in atherosclerotic patients. Little is known about the mechanisms by which ANRIL promotes CAD, except that it directly regulates the expression of ADIPOR1, VAMP3, and an orphan protein, C11ORF10. Another example of a vascular-associated lncRNA involved in disease is MALAT1 (metastasis-associated lung adenocarcinoma transcript 1; Liu et al., 2014; Michalik et al., 2014). MALAT1 is significantly upregulated during hyperglycemia and appears associated with diabetes mellitus and microvascular endothelial cell dysfunction. MALAT1 knockdown restores vessel function and alleviates inflammation in diabetic rats (Liu et al., 2014). Upon further inspection, it was found that MALAT1 accumulates in the nucleus of endothelial cells, and it is necessary for cell survival, migration, and angiogenesis in a similar fashion to lncRNA PUNISHER, as described before.

## Translational strategies

Although much remains to be learned about lncRNA biology and their mechanisms of gene regulation, lncRNAs are quickly emerging as interesting therapeutic targets in a very similar fashion to their other non-coding relatives, microRNAs. MicroRNAs have been the focus of intense translational and clinical research in the last decade due to their therapeutic promise, and a few successful examples exist and/or are close to commercialization (Kole et al., 2012; Sharma et al., 2014; Lundin et al., 2015). Most approaches are based on specific silencing of microRNA expression using short antisense oligonucleotides (ASOs) with patented derivatives resistant to cellular degradation. ASOs work on similar principles as those of microRNA-mRNA binding and degradation (Walder and Walder, 1988; Dagle et al., 1990; Braasch et al., 2002; Kurreck et al., 2002; Grünweller et al., 2003). ASOs are exciting in the context of lncRNA therapeutics because the same validated approaches as those tested for microRNAs are applicable. As a matter of fact, many of the reports described in this review have made use of ASOs to knockdown lncRNAs successfully for functional studies in mice or rats, bringing lncRNA therapeutics one step closer to the clinic. Overexpression of cardioprotective or otherwise beneficial lncRNAs is a much more complicated matter, given the length of these molecules. Efficient delivery would be complicated by the inability of the long modified transcript to cross the membrane barrier, not to mention synthesis costs and potential toxicity. As of now, alternatives would include the use of gene delivery vectors, such as engineered adeno-associated virus (AAVs), to provide exogenous expression of the desired gene (Asokan et al., 2012; Kotterman and Schaffer, 2014; Samulski and Muzyczka, 2014). Numerous clinical trials using gene therapies are underway (Ginn et al., 2013) and the European Commission already approved its first gene therapy [targeting lipoprotein lipase deficiency (LPLD)] in 2012. Additionally, the first gene therapy for children [for treatment of severe combined immunodeficiency (ADA-SCID)] was very recently approved in by the European commission. Thus therapeutic strategies aimed at lncRNA overexpression might be closer than expected.

## Conclusions and future prospects

Non-coding RNAs have emerged as critical regulators of gene expression and function. Understanding their molecular mechanisms of action is providing important novel insights into (cardiac) development, homeostasis, and disease. Judging from the number of predicted lncRNA genes, it is conceivable that future studies will clarify their role and expand our knowledge on the regulatory networks underlying cardiac development and disease. Eventually, these advances will translate into novel therapeutic approaches targeting the leading cause of death, and thus providing a substantial benefit to human healthcare.

## Author contributions

SF and AA contributed equally to the review article. They compiled the literature, conceptualized ideas, and prepared the manuscript under the supervision of LK. JH gave critical comments to the review during the preparatory phase.

### Conflict of interest statement

AA is a consultant for Jaan Biotherapeutics. The other authors declare that the research was conducted in the absence of any commercial or financial relationships that could be construed as a potential conflict of interest.
